# Differential resting-state functional connectivity patterns in functional movement disorders: Evidence for gait-disorder-specific aberrations

**DOI:** 10.1016/j.nicl.2025.103850

**Published:** 2025-07-23

**Authors:** Carl Alexander Gless, Annemarie Reincke, Anne Weissbach, Christina Bolte, Johanna Geritz, Stephan Wolff, Christof Degen-Plöger, Oliver Granert, Christian Erdmann, Svea Seehafer, Philipp J. Koch, Alexander Münchau, Norbert Brüggemann, Kirsten E. Zeuner, Jos S. Becktepe

**Affiliations:** a Department of Radiology and Neuroradiology, University of Kiel, Germany; b Department of Neurology, University of Kiel, Germany; c Institute of Systems Motor Science, Center of Brain, Behavior and Metabolism, University of Lübeck, Germany; d Center for Rare Diseases, University of Lübeck, Germany; e Institute for Health Sciences, Department of Physiotherapy, University of Lübeck, Germany; f Department of Neurology, University of Lübeck, Germany

**Keywords:** Movement disorders, Gait disorders, Neurologic, Magnetic resonance imaging, Functional, Motor network, Motor cortex, Temporoparietal junction

## Abstract

**Background:**

The clinical presentation of Functional Movement Disorders (FMD) is highly variable, encompassing gait disturbances and a wide range of hyper- and hypokinetic movement abnormalities. The neurobiological correlates distinguishing different phenotypes, particularly functional gait disorders, remain poorly understood.

**Objective:**

To investigate whether functional gait disorders are associated with specific patterns of resting-state functional connectivity that distinguish them from other FMD phenotypes.

**Methods:**

Thirty-eight FMD patients (9 with isolated gait disorders, 9 with combined gait and other motor symptoms, 20 with non-gait motor symptoms) and 20 healthy controls underwent resting-state functional MRI. Using seed-based connectivity analysis with eight bilateral regions of interest of the general, non FMD-specific motor network, we examined functional connectivity patterns across groups.

**Results:**

Seed-based functional connectivity analysis revealed decreased connectivity between the left caudate nucleus and the left temporoparietal junction across gait disorder groups compared to both non-gait disorders and healthy controls. Patients with gait disorders showed decreased interhemispheric connectivity between primary sensorimotor areas compared to non-gait disorder patients, but partially increased connectivity compared to healthy controls. Both isolated and combined gait disorder groups demonstrated characteristic alterations in premotor-sensorimotor connectivity patterns, with distinct profiles between these subgroups.

**Conclusion:**

Our findings suggest distinct neurobiological signatures in functional gait disorders compared to other functional movement phenotypes. These findings might reflect specific pathophysiological mechanisms underlying functional gait disorders, particularly involving sensory feedback integration and motor control. These results provide new insights into the neurobiological basis of different FMD phenotypes and may contribute to the development of targeted therapeutic approaches.

## Highlights

• Resting-state fMRI analysis reveals distinct neurobiological signatures in patients with functional gait disorders.
• Decreased connectivity between left caudate nucleus and left temporoparietal junction in gait disorder groups.
• New insights into neural basis of functional movement disorders may aid in developing targeted therapies.

## 1 Introduction

Functional Movement Disorders (FMD), a subset of functional neurological disorders (FND), comprise a heterogeneous group of conditions characterized by involuntary movements or involuntarily uncoordinated movements that are not attributable to known neurodegenerative or structural brain pathologies. While sharing common features, FMD present with different phenotypes, including gait disorders, hyperkinetic or hypokinetic movements such as tremor-, dystonia-, myoclonus- or parkinsonism-like movements. Despite the potential relevance of distinguishing these different phenotypes for diagnosis and treatment, their neurobiological basis is currently poorly understood.

In movement disorder specialized neurology departments, FMD accounts for 3–16 % of cases (Edwards and Bhatia, 2012, Galli et al., 2020 May 1). The most common isolated subtypes include functional tremor (21.6 %), functional weakness (18.1 %), and functional dystonia (11.8 %), as identified in an epidemiological *meta*-analysis (Lidstone et al., 2022). While isolated functional gait disorders are less common (5.7 %), they often occur in combination with other functional movement disorders. In fact, between 40 to 90 % of patients with different types of FMD may have additional functional gait disorders (Sudarsky, 2006 Jul, Gelauff et al., 2020 Jan) and may present with particularly heterogeneous clinical features. Although no consensus classification exists, a categorization into several subgroups has been proposed, including slow gait, dystonic gait, bizarre gait, astasia-abasia, and many others (Baik and Lang, 2007). While gait disorders frequently occur as an additional symptom in FMD patients, their presentation varies considerably: some patients show no gait impairment, while for others, it constitutes the sole manifestation (Lidstone et al., 2022). This clinical heterogeneity raises important questions about the distinct pathophysiological mechanisms underlying different FMD phenotypes, particularly regarding functional gait disorders with or without additional symptoms. To date, no studies have investigated these differences, with only few papers addressing the specific pathophysiological correlates of other FMD-subtypes at all (Canu et al., 2020, Piramide et al., 2022 Jun).

Concerning underlying neurobiological mechanisms of FND and FMD in general, an increasing number of studies employing functional Magnetic Resonance Imaging (fMRI) have been conducted, utilizing both task-specific and resting-state methodologies. These studies have predominantly identified and characterized abnormalities within and between motor networks and the limbic system.

Neuroimaging studies have identified several distinct functional connectivity (FC) alterations within motor networks of FMD patients.

A positive correlation has been established between the activity in the primary motor cortex (M1) and the severity of symptoms in patients with FMD (Faul et al., 2020). Additionally, FMD patients demonstrate reduced functional connectivity (FC) between the right temporoparietal junction (TPJ) and ipsilateral precentral gyrus (Maurer et al., 2016).

Further investigation revealed altered subcortical connectivity patterns in FMD patients. Specifically, enhanced FC of the right caudate nucleus to the left amygdala and bilateral postcentral gyri, alongside reduced connectivity to the left hippocampus have been demonstrated (Wegrzyk et al., 2017). This pattern potentially indicates disrupted integration of emotional and sensorimotor processing. Furthermore, patients diagnosed with functional dystonia (FD) exhibit enhanced functional connectivity between the ventromedial prefrontal cortex and left caudate nucleus, as well as the left putamen, when compared to HC (Canu et al., 2020).

Within secondary motor areas, the supplementary motor area (SMA) shows reduced FC to both the TPJ (Maurer et al., 2016) and dorsolateral prefrontal cortex (dlPFC) (Voon et al., 2011). Conversely, the SMA exhibits increased connectivity with the amygdala during emotional processing tasks (Voon et al., 2010 May, Hassa et al., 2017 Apr). Moreover, patients with fixed functional dystonia present with aberrant FC of the dorsal anterior cingulate cortex (ACC) and the SMA, distinguishing them from both healthy controls (HC) and patients with mobile functional dystonia (Canu et al., 2020). A single-subject investigation documented increased functional connectivity between the right medial prefrontal cortex and bilateral premotor cortex during bimanual alternating tapping tasks, coinciding with clinical improvement (Dogonowski et al., 2019).

Investigation of amygdala seed-based connectivity revealed enhanced connectivity with premotor cortex following therapeutic intervention. A shift in amygdala functional connectivity from ventromedial prefrontal cortex to premotor regions was documented post-treatment (Faul et al., 2020).

Beyond these FC changes in the motor network, alterations in the neural mechanisms governing sense of agency also play a crucial role in FMD pathophysiology. Sense of agency is an established pathophysiological concept in FMD research, where the right TPJ serves as a critical site for the integration of movement predictions and sensory feedback (Baizabal-Carvallo et al., 2019 Jul, Hallett et al., 2022 Jun 1). Studies have shown marked hypoactivity in the right TPJ and diminished FC between the TPJ, sensorimotor areas, and limbic regions (Voon et al., 2010). The dlPFC, another key component of the agency network, shows reduced connectivity to motor control regions, particularly the left SMA (Voon et al., 2011) and right inferior parietal cortex, along with altered responsiveness to simulated loss of control (Nahab et al., 2017).

While these studies have significantly advanced our understanding of FMD's neurobiological basis, a critical gap remains in our knowledge of phenotype-specific mechanisms. The existing literature has largely approached FMD as a homogeneous condition, with only few studies focusing on well-defined phenotypes: Studies of functional dystonia have shown reduced FC between bilateral M1 and regions such as the left dorsal ACC, SMA, dorsal posterior cingulate cortex and the precuneus (Piramide et al., 2022), while studies on “jerky and tremulous” FMD have shown alterations in default mode network activity (Marapin et al., 2021). Comparisons between “fixed” and “mobile” dystonia have identified distinct connectivity profiles, suggesting differential processing of sensory, motor, and cognitive information (Marapin et al., 2021).

The need to understand phenotype-specific mechanisms is particularly important for functional gait disorders, which present with remarkable clinical heterogeneity and often co-occur with other FMD symptoms. Despite their prevalence and impact, their unique neurobiological correlates remain largely unexplored, especially in comparison to other FMD phenotypes. Understanding these distinctions could have implications for developing more specific physio- and psychotherapeutic approaches and therapeutical imaging outcomes measures.

To address this knowledge gap, we designed a comparative cross-sectional study investigating whether functional gait disorders are associated with specific patterns of resting-state FC distinguishing them from other FMD phenotypes. Based on previous research in other FMD subtypes, particularly functional dystonia (Piramide et al., 2022), we hypothesized that while patients with functional gait disorders would share fundamental connectivity patterns in motor control networks with other FMD subtypes, they would also exhibit distinct, subtype-specific connectivity patterns within these networks.

## 2 Methods

### 2.1 Subjects

We recruited 39 patients with clinically evident FMD, diagnosed by at least two movement disorder specialists at the Departments of Neurology of the University Hospitals of Kiel and Lübeck, Germany and the Institute of Systems Motor Science, University of Lübeck. The diagnosis of FMD was confirmed through repeated evaluations before and over the course of an accompanying clinical trial, ensuring high diagnostic confidence. Exclusion criteria comprised severe neuropsychiatric or neurological comorbidities, isolated pain disorder, isolated paroxysmal functional movement disorder, isolated functional paralysis, age below 18 years, and insufficient knowledge of the German language. The severity of depressive symptoms was assessed with the Beck Depression Inventory II (BDI-II) and of anxiety symptoms using the Hospital Anxiety and Depression Scale (HADS). The patients were categorized into three groups based on their clinical presentation: (1) isolated gait disorders (iGD; n = 9), (2) combined gait disorders with other motor symptoms (cGD; n = 9), and (3) isolated motor symptoms without gait disorders (nGD; n = 21). Additionally, 20 healthy individuals served as controls (HC), with the sample size chosen to match the size of the largest FMD subgroup. Exclusion criteria for HC included any neurological, severe psychiatric, or systemic diseases that could affect resting-state FC, as well as the aforementioned exclusion criteria applied to FMD patients. All participants provided written informed consent. The study was approved by the Ethics Committees of the University Hospitals of Kiel and Lübeck and conducted in accordance with the approved guidelines.

### 2.2 Statistical analysis

Demographic and clinical characteristics were compared between groups. Normal distribution was assessed using Shapiro-Wilk test. Age was normally distributed and therefore compared between groups using one-way analysis of variance (ANOVA). Due to non-normal distributions of severity of depressive and anxiety symptoms, according to BDI-II and HADS, as well as disease duration, these variables were analyzed using Kruskal-Wallis tests. Chi-square test was used to assess gender differences. Fisher's exact test was employed for the comparison of site distribution due to small cell frequencies. Statistical significance was set at p < 0.05. Statistical analyses were performed using Python with the NumPy and SciPy.stats packages.

### 2.3 Imaging acquisition

To ensure adequate patient recruitment given the low prevalence of FMD, data collection was conducted at two sites using matched acquisition protocols. All participants underwent MRI scanning using 3 T scanners from Siemens Healthineers (Erlangen, Germany). Of the total sample, 17 patients were scanned in Lübeck on a MAGNETOM Vida system, while in Kiel, 16 patients were scanned on a MAGNETOM Vida and 6 patients on a MAGNETOM Cima.X (following a hardware upgrade at the site). All HC were scanned on the MAGNETOM Cima.X system in Kiel. To ensure compatibility of data across scanning sites and systems, we implemented identical acquisition protocols with matched sequence parameters. All acquisitions used a 64-channel head coil. Participants were scanned while continuing their regular medication.

Two sequences were acquired for resting-state FC analysis. Anatomical reference images were obtained using T1-weighted 3D sequences in sagittal orientation with the following parameters: field of view (FOV) = 256 × 256 mm^2^, isotropic voxel resolution = 1.0 × 1.0 × 1.0 mm^3^, repetition time (TR) = 1900 ms, echo time (TE) = 2.44 ms, flip angle = 9°, and 192 slices.

Functional data were acquired using echo planar imaging (EPI) blood oxygen level-dependent (BOLD) sequences in transverse orientation with: FOV = 204 × 204 mm^2^, voxel resolution = 2.4 × 2.4 × 2.4 mm^3^, TR = 1000 ms, TE = 34 ms, flip angle = 70°, 60 slices with no interslice gap, acquiring 360 volumes.

Prior to scanning, participants were familiarized with the resting-state protocol, which involved closing their eyes and avoiding focused thoughts. These instructions were repeated immediately before the EPI sequence. Participants were provided with noise protection and positioned comfortably with cushion-supported knee flexion. Patients were visually monitored throughout the scanning session to ensure absence of motor symptoms.

### 2.4 fMRI data preprocessing and statistical analysis

Analyses were performed using CONN release 22.a (Nieto-Castanon and Whitfield-Gabrieli, 2024) and SPM12 (Penny et al., 2006).

#### 2.4.1 Preprocessing

Functional and anatomical data underwent preprocessing including realignment (SPM realign & unwarp), outlier detection (framewise displacement >0.9 mm, global BOLD signal changes > 5 SD), and direct segmentation. Data were normalized to MNI space using SPM's unified segmentation algorithm with the IXI-549 template, resampled to 2 mm isotropic voxels, and smoothed with an 8 mm FWHM Gaussian kernel. No additional harmonization methods (such as ComBat) were applied to adjust for site differences. Quality assurance of the resting-state data included visual inspection of all raw images for artifacts, assessment of temporal signal-to-noise ratio, and evaluation of head motion parameters. One initially recruited patient of the nGD-group was excluded due to excessive head motion, resulting in a final sample of 38 patients and a nGD-group size of 20.

#### 2.4.2 Denoising

Denoising was performed using a standard denoising pipeline (Nieto-Castanon, 2020), including regression of white matter (20 CompCor components) and CSF timeseries (10 CompCor components), motion parameters with derivatives (12 factors), outlier scans (<206 factors), session effects with derivatives (2 factors), and linear trends (2 factors). CompCor components were estimated from eroded segmentation masks. Data were bandpass filtered between 0.008–0.09 Hz. Post-denoising effective degrees of freedom ranged from 17.7 to 51.5 (mean 50.4).

#### 2.4.3 First-level analysis

**Fig. 1:**
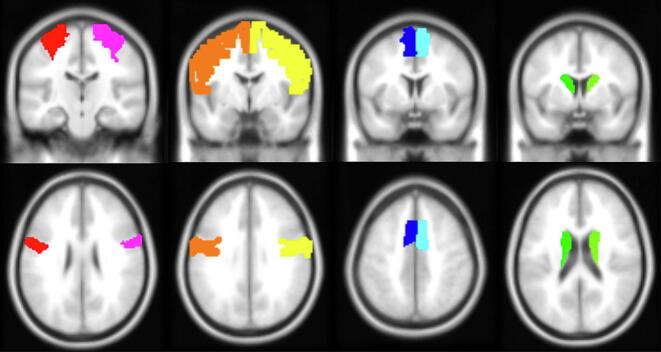
Seed Regions for the Functional Connectivity Resting-State Analysis. From left to right: primary motor cortex (red), premotor cortex (yellow), supplementary motor area (blue) and caudate nucleus (green). Definition of seed regions was performed using the WFU-Pickatlas (NeuroImaging Tools & Resources Collaboratory, Worcester, Massachusetts, USA). (For interpretation of the references to colour in this figure legend, the reader is referred to the web version of this article.)

Seed-based connectivity maps were generated using eight anatomically defined regions of interest (see Fig. 1): bilateral precentral gyrus, SMA, and caudate nucleus (from AAL atlas), and PMC (defined as Brodmann area 6 minus SMA, using WFU-Pickatlas (NeuroImaging Tools & Resources Collaboratory, Worcester, Massachusetts, USA)). ROI selection was based on their established key functions in motor control and gait: the precentral gyrus as the M1 responsible for movement execution; the SMA and PMC as secondary motor areas crucial for movement planning and coordination; and the caudate nucleus as a key subcortical structure involved in motor learning and automatization. Functional connectivity was quantified using Fisher-transformed correlation coefficients between each seed region and target voxel's BOLD timeseries, calculated using a weighted general linear model. Initial scanning volumes were down-weighted using an HRF-convolved step function to account for magnetization equilibration.

#### 2.4.4 Group-level analysis

Group-level analyses were performed using a General Linear Model (GLM) approach. First-level connectivity measures at each voxel served as dependent variables, with group membership as the independent variable. Statistical inference was performed at the cluster level using Gaussian Random Field theory. Results were thresholded using a cluster-forming threshold of p < 0.001 at the voxel level, combined with cluster-level FDR correction (p < 0.05). To account for multiple comparisons across the eight seed regions, an additional FDR correction (q < 0.05) was applied to the cluster-level p-values. Five between-group contrasts were tested: (iGD + cGD) > nGD, iGD > nGD, cGD > nGD, (iGD + cGD) > HC, and nGD > HC. All contrasts except nGD > HC were corrected for gender due to group differences in gender distribution. Age was not included as a covariate in the analysis.

#### 2.4.5 Clinical correlation of connectivity measures

Clinical symptom severity was assessed using the Psychogenic Movement Disorders Rating Scale (PMDRS) (Hinson et al., 2005) and Simplified Functional Movement Disorders Rating Scale (S-FMDRS) (Nielsen et al., 2017 Mar 11); gait sub-scores were used for analysis. Assessments were video-recorded before MRI scanning and later rated by movement disorder specialists blinded to fMRI results. For correlation analyses, we focused on two connectivity patterns that showed the most consistent and robust alterations specific to gait disorders: left caudate – middle temporal gyrus connectivity and interhemispheric precentral connectivity. Mean connectivity values were extracted from the suprathreshold clusters identified in the (iGD + cGD) > nGD contrast: left caudate seed connectivity with the left middle temporal gyrus cluster and right precentral gyrus seed connectivity with the contralateral motor cluster.

Statistical analyses were performed using Python with NumPy, SciPy, Pandas, and Statsmodels packages. We calculated Pearson correlation between connectivity and gait scores for (1) the combined patient sample, (2) gait disorder patients (iGD + cGD) and non-gait disorder patients (nGD) separately, and (3) partial correlations controlling for age and gender.

## 3 Results

### 3.1 Demographic and clinical characteristics

The patient group was predominantly female (61 %) with a mean age of 45.24 years (SD = 13.69, range = 21–64). The average disease duration was 9.64 years (SD = 15.59). The mean degree of depressive symptoms according to BDI-II was 15.57 (SD = 9.82). The mean HADS score was 14.58 (SD = 8.69). Of the nine patients with gait disorders and additional motor symptoms (cGD), six had concurrent tremor or jerks, and three presented with dystonia. Of the twenty patients without gait involvement (nGD), 13 primarily presented with tremor or myoclonus, and seven with dystonia. Notably, at least two patients displayed both phenotypes.

The HC group consisted of 20 participants (40 % female) with a mean age of 41,7 years (SD = 12,79, range = 20–60). Their mean BDI-II score was 4,75 (SD = 5.81). The mean HADS score was 5.50 (SD = 3.90). All diagnosed comorbidities were systematically recorded. Apart from depressive symptoms, conditions with potential relevance to brain connectivity included: cGD: one patient with resolved optic neuritis, iGD: one patient with migraine, nGD: one patient with migraine and one patient with resolved meningitis and resolved traumatic brain injury. Additional systemic comorbidities were present across all groups. Participants continued their regular medications during scanning. CNS-active medications were distributed across groups as follows: antidepressants (cGD: 1/9, iGD: 4/9, nGD: 4/19), neuroleptics (cGD: 1/9, iGD: 0/9, nGD: 1/19). One nGD patients took an anticholinergic, one nGD patient took gabapentin as well as an opioid. The groups did not differ significantly in antidepressant or neuroleptic use.

One-way ANOVA revealed no significant differences in age between groups (p = 0.213), with mean ages of 46.67 years for iGD, 39.11 years for cGD, 47.35 years for nGD, and 41.70 years for HC. Gender distribution differed significantly between groups (χ^2^ test, p = 0.033), with higher female proportions in both gait disorder groups (iGD and cGD: 77.8 % each) compared to nGD (45.0 %) and HC (40.0 %). Consequently, gender was included as a covariate in analyses of groups with uneven gender distributions. Overall, female participants (n = 31, mean age = 42.4 years) did not differ significantly in age from male participants (n = 27, mean age = 45.9 years; *t*-test, p = 0.33). Within each group, no significant age differences were found between genders (HC: p = 0.62, iGD: p = 0.67, cGD: p = 0.30, nGD: p = 0.63). Degree of depressive symptoms differed significantly between groups (Kruskal-Wallis H = 17.46, p < 0.001), with healthy controls showing lower BDI-II scores. Similarly, HADS scores differed significantly between groups (Kruskal-Wallis H = 17.83, p < 0.001). Among patients, severity of symptoms of depression and anxiety was similar across groups (BDI-II: H = 1.33, p = 0.51, HADS: H = 3.21p = 0.20). Disease duration showed no significant differences between patient groups (H = 1.727, p > 0.05). Demographic and clinical characteristics are summarized in Table 1*.*

**Table 1:** Demographics and clinical characteristics of patients and controls. iGD: isolated gait disorders, cGD: combined gait disorders, nGD: no gait disorders HC: healthy controls, BDI = beck depression inventory, HADS: Hospital Anxiety and Depression Scale. Values are rounded to the second decimal. Values are presented as mean ± standard deviation unless otherwise specified. ^1^One-way ANOVA. ^2^Chi-square test. ^3^Kruskal-Wallis test.

	iGD (n = 9)	cGD (n = 9)	nGD (n = 20)	HC (n = 20)	p-value
Age (years)	46.67 ± 12.10	39.11 ± 11.47	47.35 ± 15.40	41.70 ± 12.79	0.21^1^
Gender (f/m)	7/2	7/2	11/9	8/12	0.03^2^
Disease duration (years)	6.33 ± 7.00	11.11 ± 11.72	8.05 ± 16.60	−	0.45^3^
BDI	18.00 ± 9.77	12.89 ± 9.78	15.57 ± 9.82	4.75 ± 5.81	<0.01^3^
HADS	15.56 ± 9.04	10.78 ± 9.31	15.40 ± 8.65	5.50 ± 3.90	<0.01^3^

Of the iGD group, 5 patients were scanned in Kiel and 4 in Lübeck, showing a balanced distribution. The distribution of the remaining patients across sites differed between patient groups with 2 patients of the cGD group scanned in Kiel and 7 in Lübeck, while the nGD group had 14 patients in Kiel and 6 in Lübeck. Fisher's exact test revealed a marginally significant difference in site distribution between patient groups (p = 0.047). All healthy controls (n = 20) were scanned in Kiel.

### 3.2 Functional connectivity analysis

Seed-based functional connectivity analysis revealed distinct patterns of altered connectivity in primary and secondary motor areas when comparing patients with functional gait disorders to both patients without gait disorders and healthy controls. Most notably, we observed consistent alterations in interhemispheric sensorimotor connectivity and subcortical-temporoparietal interactions. The detailed findings for each contrast are described below and summarized in Table 2.

**Table 2:** Significant Clusters in Group Comparisons. Size p-FDR shows the significance at the individual cluster level. Seed q-FDR indicated significance after correction for multiple seed regions. iGD: isolated gait disorders, cGD: combined gait disorders, nGD: no gait disorders, HC: healthy controls. PreCG: precentral gyrus, PostCG: postcentral gyrus, SMA: supplementary motor area, PMC: premotor cortex, L: left, R: right. FDR-correctet p-value of cluster size is rounded to the fourth decimal. * AAL regions listed based on cluster overlap, as peak coordinates are not within defined AAL regions. Results of the (iGD + cGD > nGD) contrast are presented visually in Fig. 2, Fig. 3.

Contrast	Seed Region	AAL Region of Peak Voxel	Direction of Change	Cluster Size	Peak MNI Coordinates			Size p-FDR	Seed q-FDR
					x	y	z		
iGD + cGD > nGD	PreCG L	−	−	−	−			−	−
	PreCG R	Postcentral Gyrus L	↓	171	−46	−30	58	0.0009	0.0076
		Pallidum L / Caudate L*	↑	123	−4	0	0	0.0038	0.0248
		Precentral Gyrus L	↓	68	−60	8	34	0.0416	0.0481
	SMA L	−	−	−	−			−	−
	SMA R	−	−	−	−			−	−
	Caudate L	Middle Temporal Gyrus L	↓	82	−56	−60	16	0.0167	0.0449
	Caudate R	−	−	−	−			−	−
	PMC L	Precentral Gyrus L	↓	242	−60	8	36	0.0001	0.0017
		Postcentral Gyrus L	↓	134	−52	−6	52	0.0045	0.0248
	PMC R	Postcentral Gyrus L	↓	135	−62	−2	30	0.0051	0.0248
		Postcentral Gyrus R	↓	93	32	–32	44	0.0185	0.0449
		Insula R	↓	66	40	2	12	0.0465	0.0481
		Heschl’s Gyrus R	↓	61	46	−20	34	0.0465	0.0481
		Middle Frontal Gyrus R	↑	59	32	46	42	0.0465	0.0481
iGD > nGD	PreCG L	−	−	−	−			−	−
	PreCG R	Postcentral Gyrus L	↓	75	−58	−12	46	0.0467	0.0481
	SMA L	−	−	−	−			−	
	SMA R	Inferior Temporal Gyrus R	↓	80	48	−36	−24	0.0271	0.0449
	Caudate L	Middle Temporal Gyrus L	↓	69	−56	−62	16	0.0202	0.0449
		Middle Temporal Gyrus L	↓	65	−50	−26	−10	0.0202	0.0449
	Caudate R	−	−	−	−			−	
	PMC L	Inferior Parietal Gyrus L	↓	76	−58	−20	50	0.0430	0.0481
	PMC R	−	−	−	−			−	
cGD > nGD	PreCG L	−	−	−	−				
	PreCG R	−	−	−	−				
	SMA L	Superior Frontal Gyrus R	↓	78	20	28	38	0.0259	0.0449
	SMA R	−	−	−	−				
	Caudate L	−	−	−	−				
	Caudate R	Middle Temporal Gyrus R	↓	168	52	−60	20	0.0001	0.0017
		Superior Frontal Gyrus R	↓	61	18	40	56	0.0244	0.0449
	PMC L	Precentral Gyrus R	↓	81	58	8	34	0.0281	0.0449
		Precentral Gyrus L	↓	62	−58	8	38	0.0417	0.0481
	PMC R	−	−	−	−			−	
iGD + cGD > HC	PreCG L	−	−	−	−			−	
	PreCG R	Middle Temporal Gyrus L*	↑	180	−62	−62	12	0.0002	0.0023
		Middle Frontal Gyrus R	↑	78	40	6	42	0.0172	0.0449
		Rolandic Operculum R	↓	68	66	−2	12	0.0214	0.0449
		Precentral Gyrus L	↑	63	−38	0	42	0.0221	0.0449
	SMA L	−	−	−	−			−	−
	SMA R	−	−	−	−			−	−
	Caudate L	Middle Temporal Gyrus L	↓	60	−58	−58	12	0.0499	0.0499
	Caudate R	−	−	−	−			−	−
	PMC L	−	−	−	−			−	−
	PMC R	Precentral Gyrus L	↑	70	−38	4	42	0.0326	0.0449
nGD > HC	PreCG L	Superior Frontal Gyrus L	↑	118	−16	8	60	0.0094	0.0393
	PreCG R	Precuneus R	↑	118	14	−50	46	0.0104	0.0393
	SMA L	−	−	−	−			−	−
	SMA R	Not labeled (Temporal Lobe White Matter)	↓	85	−26	−46	16	0.0330	0.0449
		Superior Temporal Gyrus R	↑	76	50	−10	4	0.0330	0.0449
		Supramarginal Gyrus L	↑	73	−56	−34	28	0.0330	0.0449
	Caudate L	−	−	−	−			−	−
	Caudate R	Caudate L*	↑	77	−24	28	14	0.0314	0.0449
	PMC L	−	−	−	−			−	−
	PMC R	Middle Temporal Gyrus L	↑	89	−52	2	−14	0.0413	0.0481

#### 3.2.1 Gait disorder groups versus non-gait disorders (iGD + cGD > nGD)

Patients with gait disorders showed decreased FC between the right precentral gyrus and two clusters encompassing the left pre- and postcentral gyri, compared to patients without gait disorders. Additionally, decreased FC was observed between both premotor cortices and their corresponding ipsilateral primary sensorimotor areas. The left caudate nucleus showed lower FC with the posterior part of the left middle temporal gyrus at the left temporoparietal junction.

**Fig. 2:**
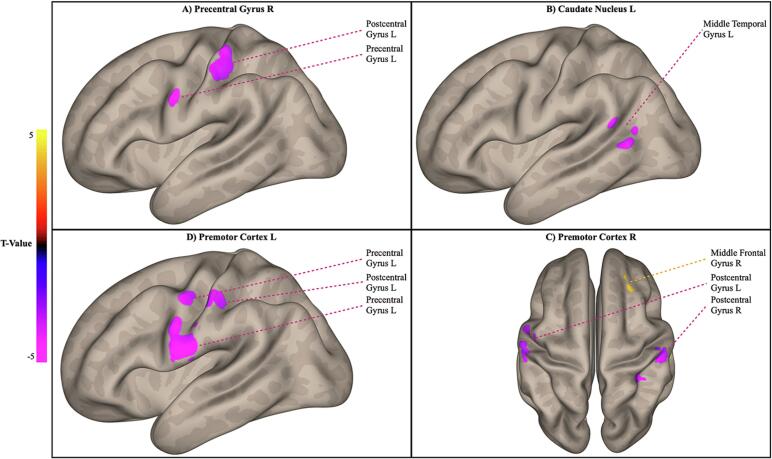
Seed-based functional connectivity differences between patients with and without functional gait disorders (iGD + cGD > nGD). A-C) Lateral views of the left hemisphere showing significant connectivity changes for the indicated seed regions. D) Superior view demonstrating connectivity changes for the right premotor cortex. Statistical maps are thresholded at p < 0.001 at voxel level to form clusters and FDR-corrected at p < 0.05 at cluster-size level. For detailed statistical results, see Table 1, Section 1.

**Fig. 3:**
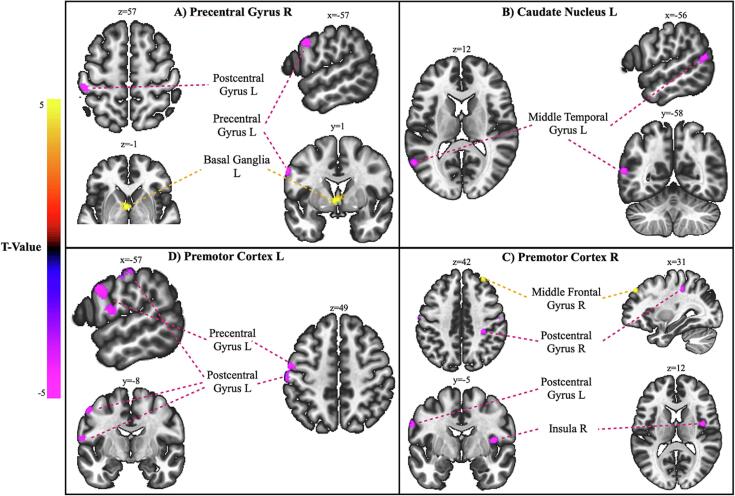
Seed-based functional connectivity differences between patients with and without functional gait disorders (iGD + cGD > nGD) showing significant connectivity changes for the indicated seed regions. Statistical maps are thresholded at p < 0.001 at voxel level to form clusters and FDR-corrected at p < 0.05 at cluster-size level. Coordinates are displayed in MNI-space. For detailed statistical results, see Table 1, Section 1.

Altogether, patients with gait disorders exhibited reduced FC across interhemispheric sensorimotor areas and between the left caudate and TPJ, compared to patients with no gait disorders. Fig. 2 and Fig. 3 summarize the main findings of this contrast.

#### 3.2.2 Isolated gait disorders versus non-gait disorders (iGD > nGD)

Patients with isolated gait disorders exhibited decreased FC between the right precentral gyrus and a cluster comprising the left postcentral gyrus. The reduced FC between the left caudate and left middle temporal gyrus / temporoparietal junction area was also preserved in this contrast.

Unique to this subgroup, patients showed lower FC between the right SMA and the right inferior temporal gyrus. While no alterations in FC between PMC and primary sensorimotor cortices were observed, reduced FC between the left PMC and left inferior parietal cortex emerged as a distinct finding.

In summary, patients with isolated gait disorders show decreased FC between the bilateral precentral gyri, between the right SMA and right inferior temporal gyrus and between the left caudate nucleus and left TPJ compared to patients with no gait disorders.

#### 3.2.3 Combined gait disorders versus non-gait disorders (cGD > nGD)

Patients with combined gait disorders displayed no significant FC alterations between left and right primary sensorimotor areas. However, they exhibited reduced FC between the left SMA and right superior frontal gyrus, a pattern unique to this contrast. The right caudate showed reduced FC to the right posterior middle temporal and superior frontal gyri, paralleling the contralateral findings in other contrasts. Consistent with the combined group contrast (iGD + cGD > nGD), reduced FC between the left PMC and bilateral precentral gyri was observed.

Overall, compared to non-gait disorder patients, those with combined gait disorders demonstrated decreased connectivity primarily in frontal-motor and caudate-temporal pathways.

#### 3.2.4 Gait disorders versus healthy controls (iGD + cGD > HC)

In contrast to the comparison with non-gait disorder patients, gait disorder patients exhibited increased FC between the right precentral gyrus and the left precentral gyrus, compared to healthy controls. However, decreased FC was observed between the right precentral gyrus and a cluster peak in the right rolandic operculum, extending into the right postcentral gyrus.

Additionally, they demonstrated increased FC between the right precentral gyrus and left middle temporal and right middle frontal gyri.

The reduced FC between left caudate and left middle temporal gyrus / temporoparietal junction aligned with previous contrasts. Notably, the increased FC between right PMC and left precentral gyrus contrasted with the decreased FC observed in earlier comparisons.

In brief, compared to HC, patients with gait disorders displayed both increased interhemispheric sensorimotor and decreased connectivity between the caudate and temporoparietal regions. The latter finding is consistent with that observed when comparing patients with gait disorders with non-gait disorder patients.

#### 3.2.5 Non-gait disorders versus healthy controls (nGD > HC)

Patients without gait disorders exhibited distinct FC patterns compared to those with gait disorders. The left precentral gyrus showed increased FC with the left superior frontal gyrus, while the right precentral gyrus showed increased FC with the right precuneus. The right SMA demonstrated increased FC with the right superior temporal and the left supramarginal gyri, and the right PMC showed increased FC with the left middle temporal gyrus.

In summary, compared to HC, non-gait disorder patients show predominantly increased connectivity in frontoparietal and temporoparietal networks, a pattern distinct from the connectivity alterations observed in gait disorder patients.

### 3.3 Connectivity-symptom correlations

In the combined patient sample, both connectivity measures showed significant negative correlations with gait symptoms: left caudate-temporal connectivity (PMDRS gait: r = −0.52, p = 0.001; S-FMDRS gait: r = −0.37, p = 0.029) and interhemispheric precentral connectivity (PMDRS gait: r = −0.69, p < 0.001; S-FMDRS gait: r = −0.64, p < 0.001). These correlations remained significant after controlling for age and gender (caudate-temporal: PMDRS gait r = −0.52, p = 0.001; S-FMDRS gait r = -0.37, p = 0.031; precentral: PMDRS gait r = −0.68, p < 0.001; S-FMDRS gait r = −0.63, p < 0.001).

However, when examined within groups, neither gait disorder patients (iGD + cGD) nor non-gait disorder patients (nGD) showed significant connectivity-symptom correlations.

## 4 Discussion

Our analysis revealed three main patterns of altered FC in functional gait disorders: (1) consistently decreased connectivity between subcortical and temporoparietal regions, specifically between the left caudate nucleus and left posterior middle temporal gyrus; (2) distinct patterns of interhemispheric sensorimotor connectivity, characterized by decreased FC between bilateral pre- and postcentral gyri compared to non-gait disorder patients but partially increased connectivity compared to healthy controls; and (3) characteristic alterations in premotor-sensorimotor connectivity patterns, which differed between isolated and combined gait disorder phenotypes. These findings suggest differences in motor network organization between functional gait disorders and other functional movement disorder phenotypes, potentially reflecting distinct pathophysiological mechanisms.

### 4.1 Subcortical-temporal connectivity in functional gait disorders

The consistent finding of decreased connectivity between the left caudate nucleus and the left posterior middle temporal gyrus across our analyses reflects an alteration in motor-parietotemporal integration in functional gait disorders. The caudate nucleus plays a crucial role in motor learning and automatization of movement sequences (Choi et al., 2019). A correlation between abnormal caudate shape and deficits in gait and balance has been shown (Macfarlane et al., 2015). The cluster of decreased connectivity we observed in the posterior part of the left middle temporal gyrus extends into the left TPJ. Notably, previous literature on FMD has predominantly implicated the right TPJ in abnormal self-agency (Maurer et al., 2016 Aug 9, Voon et al., 2010 Jan 19, Baek et al., 2017 Jul), while our findings point to left-lateralized connectivity changes. This lateralization pattern was observed based on the anatomical location of significant clusters identified in whole-brain analyses rather than on the results of a formal statistical test of hemispheric differences. According to Mueller et al., the left TPJ has stronger connectivity with executive control networks (Mueller et al., 2022). In the context of functional gait disorders, this might reflect problems with motor execution rather than the self-agency impairments typically associated with other FMD phenotypes. Our finding of disrupted connectivity between the caudate nucleus and the TPJ might therefore represent impaired integration of automated motor programs with sensory feedback and agency attribution, processes that are particularly crucial for maintaining automatic / fluid gait patterns. The consistency of this finding across our gait disorder groups, regardless of additional symptoms, suggests that it may be a distinctive neurobiological feature of functional gait disorders.

### 4.2 Interhemispheric sensorimotor integration

Our observation of decreased interhemispheric connectivity between bilateral pre- and postcentral gyri in gait disorder patients compared to non-gait FMD patients, but partially increased connectivity compared to healthy controls, is intriguing. Previous studies have shown that successful gait requires precise coordination between bilateral sensorimotor regions (Frost et al., 2015, Jacobsen et al., 2024, MacKinnon, 2018). The bidirectional alteration in interhemispheric connectivity (decreased vs nGD, increased vs HC) appears specific to gait disorders rather than representing a general FMD-related mechanism. While non-gait FMD patients show increased connectivity in different networks (primarily involving frontal and precuneus regions), gait disorder patients specifically show altered connectivity in bilateral sensorimotor regions. This pattern might reflect the unique demands of bipedal locomotion, where precise temporal coordination between hemispheres is crucial for maintaining balanced gait. The pattern of interhemispheric connectivity, especially the fact of increased FC compared to HC, shows notable complexity. While its full implications require further investigation, the overall pattern of altered sensorimotor integration appears specific for functional gait disorders.

### 4.3 Role of premotor-sensorimotor networks

The distinct patterns of premotor-sensorimotor connectivity between isolated and combined gait disorders provide new insights into the neurobiological basis of FMD phenotype heterogeneity. Our finding of reduced connectivity between premotor cortices and their ipsilateral primary sensorimotor areas in both gait disorder groups aligns with previous work showing altered premotor function in FMD (Voon et al., 2011). However, the specific patterns differed between isolated and combined phenotypes, suggesting that additional motor symptoms might influence network reorganization.

This finding has particular relevance for understanding the hierarchical organization of motor control in FMD. The premotor cortex, traditionally viewed as crucial for movement planning and sensory guidance of movement (Kantak et al., 2011 Sep 16, Ohbayashi, 2021), shows altered connectivity patterns that might reflect disrupted motor planning processes. Specifically, these alterations could represent impaired integration of external stimuli during movement control, potentially leading to an overreliance on internal signals. This interpretation could provide a neurobiological rationale for externalization techniques in physiotherapy, where patients are guided to focus on external stimuli for movement control rather than internal sensations. Reduced FC between right M1 and PMC/SMA in functional dystonia patients, suggesting that inadequate top-down regulation by premotor areas over primary motor regions may contribute to involuntary motor outputs, has been shown (Piramide et al., 2022). As highlighted in a recent review, such alterations in motor networks, particularly involving top-down regulation from frontal areas, represent a key pathophysiological component in FMD (Roelofs et al., 2019). Our observation that connectivity patterns differed between isolated and combined phenotypes suggests that additional motor symptoms might influence network reorganization, supporting the concept of phenotype-specific neurobiological signatures in FMD.

### 4.4 Relationship between connectivity patterns and clinical symptoms

The analyses of correlation between gait symptom severity and resting-state functional connectivity values showed significant associations in the combined patient sample (patients with and without gait disorders) but not within individual groups. This pattern suggests the connectivity differences may be more related to phenotype categories than to symptom severity per se.

The absence of within-group correlations could have several explanations. The connectivity alterations might represent a phenotypic characteristic of functional gait disorders without directly determining symptom severity. Other factors – psychological, environmental, or compensatory – could modulate clinical presentation, consistent with the multifactorial model of FMD (Voon et al., 2016).

Alternatively, these connectivity patterns might be associated with but not causally related to gait symptoms. They could reflect broader network reorganization in response to having functional gait disorders, represent predisposing neural patterns, or even emerge as consequences rather than causes of altered movement patterns.

It is also possible that symptom severity relates to neural mechanisms not captured by our resting-state analysis, such as dynamic network interactions during movement.

Given our sample size limitations, these interpretations remain speculative and warrant further investigation in larger studies.

### 4.5 Clinical implications

Our findings provide valuable insights that may benefit future diagnostic and therapeutic approaches. The observed network alterations, particularly in sensorimotor integration and temporoparietal-subcortical connectivity, suggest potential targets for novel physiotherapeutic interventions and could serve as paraclinical markers to evaluate therapeutic efficacy. For instance, treatment protocols might benefit from explicitly addressing proprioceptive feedback and bilateral motor coordination during movement exercises. The different connectivity patterns observed in isolated versus combined gait disorders further suggest that therapeutic approaches may need to be tailored based on symptom complexity. For isolated gait disorders, interventions may focus primarily on reestablishing normal interhemispheric sensorimotor integration, where bilateral coordination exercises using external cues could be thinkable, whereas patients with combined symptoms may benefit from a more comprehensive approach that simultaneously addresses gait and additional motor symptoms.

### 4.6 Limitations

It is important to take into account that resting-state FC measures temporal correlation between BOLD signals rather than the levels of neural activity levels directly. Therefore, our findings of increased or decreased connectivity should be interpreted as altered temporal synchronization between regions rather than evidence of hyper-or hypoactivity. However, this is an inherent characteristic of resting-state FC analyses, not a specific limitation to our study.

Additionally, several limitations specific to our study should be considered when interpreting our results. The sample size, particularly in the gait disorder subgroups (n = 9 each), was relatively small, although comparable to previous FMD neuroimaging studies given the low prevalence of these disorders. Data collection took place at two sites using different MRI scanners, which was necessary to ensure adequate recruitment. The distribution of patients between sites showed modest differences across patient groups (p = 0.047), though this effect was only marginally significant. Notably, the iGD group showed a balanced distribution between sites (5 in Kiel, 4 in Lübeck). However, all healthy controls were scanned in Kiel, so while we implemented matching acquisition protocols to minimize site-related effects, we cannot completely rule out that some of the observed group differences might be partially influenced by site effects, particularly those involving comparisons with healthy controls. There was a notable gender imbalance between the groups, with a higher proportion of women in both gait disorder groups. Although we controlled for gender in our analyses, this imbalance may reflect a true demographic characteristic of functional gait disorders rather than a methodological limitation. Additionally, our study sample was restricted to FMD patients without severe comorbidities, which may not fully represent the typical FMD population. Also, the classification of FMD phenotypes is inherently challenging due to the frequent co-occurrence of different symptoms, as demonstrated in our sample. This clinical heterogeneity, although characteristic of FMD, may have influenced our group comparisons. Furthermore, while participants continued their regular medications during scanning and presented with various comorbidities, the similar distribution of both CNS-active drugs and these conditions across groups makes them unlikely to explain our phenotype-specific findings. Additionally, our connectivity-symptom correlations in the combined sample appear driven by group differences rather than associations with symptom severity, and our cross-sectional design cannot determine whether connectivity alterations cause, result from, or merely accompany functional gait symptoms.

## 5 Conclusion

Our study reveals distinct neurobiological signatures in functional gait disorders, characterized by three key patterns: decreased connectivity between subcortical and temporoparietal regions, altered interhemispheric sensorimotor integration, and phenotype-specific changes in premotor-sensorimotor networks. These findings not only increase our understanding of the neurobiological mechanisms underlying different phenotypes of functional movement disorders, but also suggest potential targets for therapeutic intervention. The identification of these disorder-specific patterns may guide the development of more targeted treatment approaches and provides a foundation for future research into objective markers for diagnosis and treatment monitoring in functional gait disorders.

## CRediT authorship contribution statement

**Carl Alexander Gless:** Investigation, Conceptualization, Visualization, Data curation, Writing – original draft, Methodology, Formal analysis. **Annemarie Reincke:** Resources, Data curation, Investigation, Project administration. **Anne Weissbach:** Funding acquisition, Project administration, Investigation, Conceptualization, Supervision, Writing – review & editing, Methodology. **Christina Bolte:** Writing – review & editing, Conceptualization, Project administration, Formal analysis, Investigation. **Johanna Geritz:** Resources, Project administration, Data curation, Writing – review & editing, Investigation. **Stephan Wolff:** Methodology, Investigation, Resources, Data curation, Writing – review & editing. **Christof Degen-Plöger:** Investigation, Methodology. **Oliver Granert:** Software, Formal analysis, Supervision, Visualization, Methodology, Writing – review & editing, Resources. **Christian Erdmann:** Methodology, Data curation, Resources, Investigation. **Svea Seehafer:** Methodology, Visualization, Formal analysis. **Philipp J. Koch:** Writing – review & editing, Project administration, Investigation. **Alexander Münchau:** Supervision, Funding acquisition, Writing – review & editing, Methodology, Resources, Conceptualization. **Norbert Brüggemann:** Writing – review & editing, Funding acquisition, Methodology, Supervision, Resources, Conceptualization. **Kirsten E. Zeuner:** Writing – review & editing, Writing – original draft, Resources, Funding acquisition, Project administration, Investigation, Supervision, Methodology, Conceptualization. **Jos S. Becktepe:** Writing – original draft, Methodology, Supervision, Funding acquisition, Writing – review & editing, Conceptualization.

## Funding

This work was supported by the Deutsche Forschungsgemeinschaft (DFG, grant number WE 5919/4-1).

## Declaration of competing interest

The authors declare the following financial interests/personal relationships which may be considered as potential competing interests: CAG was supported by the Faculty of Medicine, Christian-Albrechts-University Kiel.

NB received honaria from Abbott, Abbvie, Biogen, Esteve, Ipsen, Merz, Teva, Zambon and was supported by the DFG (BR4328.2-2, GRK1957), the Michael J Fox Foundation, and the EU Joint Programme − Neurodegenerative Disease Research (JPND).

KEZ has received research support from Strathmann and the German Research Council. She reports speaker’s honoraria from Bayer Vital GmbH, BIAL, AbbVie, Alexion, Allergan and Merz outside the submitted work. She has served as a consultant and received fees from Merz, Ipsen, Alexion, Bial and the German Federal Institute for Drugs and Medical Devices (BfArM).

JSB has received consultancies/advisory board fees from Jazz Pharmaceuticals and Neuraxpharm, research support from Strathmann and speaker’s honoraria from Ipsen and Stadapharm.

## Data Availability

The data that has been used is confidential.
